# The Use of Percent Change in RR Interval for Data Exclusion in Analyzing 24-h Time Domain Heart Rate Variability in Rodents

**DOI:** 10.3389/fphys.2019.00693

**Published:** 2019-06-06

**Authors:** Emma Karey, Shiyue Pan, Amber N. Morris, Donald A. Bruun, Pamela J. Lein, Chao-Yin Chen

**Affiliations:** ^1^Department of Pharmacology, School of Medicine, University of California, Davis, Davis, CA, United States; ^2^Department of Molecular Biosciences, School of Veterinary Medicine, University of California, Davis, Davis, CA, United States

**Keywords:** electrocardiogram, rMSSD, SDNN, HRV, tachogram, Lorenz plot, ROC, telemetry

## Abstract

While epidemiological data support the link between reduced heart rate variability (HRV) and a multitude of pathologies, the mechanisms underlying changes in HRV and disease progression are poorly understood. Even though we have numerous rodent models of disease for mechanistic studies, not being able to reliably measure HRV in conscious, freely moving rodents has hindered our ability to extrapolate the role of HRV in the progression from normal physiology to pathology. The sheer number of heart beats per day (>800,000 in mice) makes data exclusion both time consuming and daunting. We sought to evaluate an RR interval exclusion method based on percent (%) change of adjacent RR intervals. Two approaches were evaluated: % change from “either” and “both” adjacent RR intervals. The data exclusion method based on standard deviation (SD) was also evaluated for comparison. Receiver operating characteristic (ROC) curves were generated to determine the performance of each method. Results showed that exclusion based on % change from “either” adjacent RR intervals was the most accurate method in identifying normal and abnormal RR intervals, with an overall accuracy of 0.92–0.99. As the exclusion value increased (% change or SD), the sensitivity (correctly including normal RR intervals) increased exponentially while the specificity (correctly rejecting abnormal RR intervals) decreased linearly. Compared to the SD method, the “either” approach had a steeper rise in sensitivity and a more gradual decrease in specificity. The intersection of sensitivity and specificity where the exclusion criterion had the same accuracy in identifying normal and abnormal RR intervals was 10–20% change for the “either” approach and ∼ 1 SD for the SD-based exclusion method. Graphically (tachogram and Lorenz plot), 20% change from either adjacent RR interval resembled the data after manual exclusion. Finally, overall (SDNN) and short-term (rMSSD) indices of HRV generated using 20% change from “either” adjacent RR intervals as the exclusion criterion were closer to the manual exclusion method with lower subject-to-subject variability than those generated using the 2 SD exclusion criterion. Thus, 20% change from “either” adjacent RR intervals is a good criterion for data exclusion for reliable 24-h time domain HRV analysis in rodents.

## Introduction

The concept of using respiration-related changes in heart beat (respiratory sinus arrhythmia) as a marker for cardiac vagal regulation was first introduced four decades ago (Katona and Jih, 1975; Hedman et al., 1995). Over the past two decades, heart rate variability (HRV) has become a clinical tool for assessing autonomic function and risks for cardiac events (Villareal et al., 2002). Attenuated HRV is associated with myriad pathologies, and reduced HRV has also been shown to be an independent risk factor for cardiac events including arrhythmias and sudden cardiac death (Kleiger et al., 2005; Billman, 2011).

HRV parameters can be derived with time domain, frequency domain, and non-linear dynamic analysis techniques (Rajendra Acharya et al., 2006; Billman, 2011). Regardless of the parameters used to assess HRV, it is crucial that abnormal RR intervals (ectopic beats, noise, artifacts, etc.,) are excluded in the analysis for accurate HRV measures (Malik et al., 1996). Because including abnormal beats in the analysis can compromise the reliability of HRV measures, data exclusion is an essential part of the HRV analysis (Lippman et al., 1994; Peltola, 2012). This is feasible with short-term recordings in humans under standardized conditions in which the recorded signals typically contain minimal artifacts (Lippman et al., 1994; Peltola, 2012). With longer recording periods and/or recordings from conscious and freely moving subjects, abnormal beats and artifacts become common in the data set, which makes identifying abnormal RR intervals a challenging task. This is particularly true for recordings from mice, in part due to their size; changes in posture and activity can profoundly alter the relative position of the two electrocardiogram (ECG) leads, which alters the quality of ECG signals, as well as introduces electromyography noise or creates non-physiological noise from lead movements. In addition, the sheer number of heart beats in 24 h (>800,000 beats in mice and >400,000 beats in rats) makes data exclusion time consuming and daunting. Based on our previous studies, on average, a 24-h ECG recording from a mouse requires approximately 1 week to visually inspect all RR intervals and manually exclude abnormal RR intervals (Chen et al., 2008; Pham et al., 2009).

Thus, applying the strict criterion of including only normal-to-normal RR intervals for HRV analysis manually is not practical in HRV analysis of rodent ECG recordings. It is thus necessary to strike a balance between accuracy in identifying abnormal RR intervals and the time required to do so. Thireau and colleagues presented a simple two standard deviations (2 SD) exclusion method in which RR intervals outside of the 95% confidence intervals (mean ± 2 SD) were excluded from data analysis (Thireau et al., 2008). Albeit not perfect, this numeric cut off based on the 95% confidence intervals makes it feasible to process 24-h mouse recordings within a reasonable time frame.

The purpose of this paper is to evaluate a different RR interval exclusion method based on percent (%) change of adjacent RR intervals. The gold standard of manual exclusion was used as the baseline for comparison. Mouse and rat ECG recordings with varying types of noise adulterating the ECG signals were used to test the efficacy of each exclusion method.

## Materials and Methods

All protocols were approved by the University of California, Davis Institutional Animal Care and Use Committee in compliance with the Animal Welfare Act and Public Health Service Policy on Humane Care and Use of Laboratory Animals. We selected three mouse recordings that represent different degrees of artifacts: recording 1 (M1) had a large quantity of erroneous extra marks (non-normal R waves) and missed marks (unmarked normal R waves) throughout the 24-h recording; recording 2 (M2) had mostly erroneous extra marks, with most of the erroneous marks in the dark cycle and with long stretches of abnormal RR intervals; and recording 3 (M3) had relatively fewer artifacts throughout the recordings (mostly ideal ECG waveforms and correct R wave marks).

Similarly, three rat recordings representing different degrees of artifacts were used to evaluate the exclusion methods. Among the three rats, recording 1 (R1) contained the least amount of noise and artifacts while rat recording 2 (R2) had the most noise and artifacts in the recordings.

### Telemetry Implant (Mouse)

Male C57BL/6J mice (8 weeks old from The Jackson Lab, Sacramento, CA) were anesthetized with isoflurane (5% induction, 1.5–3% maintenance). The criteria for adequacy of anesthesia included the following: (1) lack of eye blink reflex, (2) no whisker movement, (3) lack of paw pinch withdraw, and (4) no irregular or sudden changes in breathing frequency. A mouse blood pressure (BP) + ECG telemetry device (HD-X11, Data Sciences International, St. Paul, MN, United States) was implanted subcutaneously in the left side of body via a small midline incision at the ventral neck region. The tip of the arterial catheter was placed in the aortic arch through the left carotid artery and both ECG leads were tunneled subcutaneously to obtain a lead II configuration. The negative lead was secured to the upper right pectoral muscle wall, and the positive lead was sutured just medial of the xiphoid process. Mice were given Buprenex (0.05 mg/kg) subcutaneously prior to surgery and twice daily post-op for 2 days for pain control.

### Telemetry Implant (Rat)

Male Sprague Dawley rats (8 weeks old from Charles River Laboratories, Hollister, CA) were anesthetized with isoflurane (5% induction, 1.5–3% maintenance). The criteria for adequacy of anesthesia were the same as those for the mice. A rat BP/ECG telemetry device (HD-S11, Data Sciences International, St. Paul, MN, United States) was implanted via midline laparotomy. The arterial catheter was inserted into the abdominal aorta and secured with a drop of Vetbond surgical glue and a nitrocellulose patch. The transmitter body was secured to the abdominal muscle wall with 5-0 non-absorbable suture. Both ECG leads were tunneled through the abdominal muscle wall caudoventrally and then tunneled subcutaneously for a lead II configuration. Rats were given Buprenex (0.05 mg/kg) subcutaneously prior to surgery and twice daily post-op for 2 days for pain control.

### Recording Protocols

All animals were housed individually on a 12-h dark-light cycle with food and water available *ad libitum* (mouse housing facility: temperature 69 ± 4°F, 60 ± 15% humidity, rat housing facility: 73 ± 2°F, 48 ± 5% humidity). Recordings were performed in a dedicated animal housing room in which no personnel entered during the recording period. ECGs were recorded at 4 kHz and BP was recorded at 500 Hz with Ponemah software (Data Sciences International, St Paul, MN, United States). For mice, 24-h continuous BP/ECG recordings were obtained 10–14 weeks after implant surgery. For rats, 24-h continuous BP/ECG recordings were obtained 3 weeks after implant surgery.

### Marking ECG R-Waves

The BP waveform was used as a reference for heart beats with regular cardiac contractions. R waves were marked with Ponemah analysis attributes as listed in Table 1. In addition, for mice, any RR intervals longer than 400 ms (HR < 150 bpm) were excluded using Data Insights software (Data Sciences International, St. Paul, MN, United States). For rats, RR intervals longer than 600 ms (HR < 100 bpm) were excluded. These resulting data sets were referred to as the “initial” data sets and subsequently subjected to three different RR interval exclusion methods (manual, % change-based, and SD-based) as described below.

**TABLE 1:** Ponemah Analysis Attribute settings used for marking R waves in ECG.

**Attribute**	**Mice setting**	**Rat setting**
QRS detection threshold	25%	25%
Minimum R deflection	0.03–0.25 mV	0.3 mV
Maximum heart rate	1500 bpm	1200 bpm
Minimum heart rate^*^	400 bpm	200 bpm
Peak bias	20%	20%

For QRS detection threshold, 25% of the largest rectified derivative signal within a QRS segment is used for identifying potential R waves. Minimum R deflection is the threshold voltage for R wave detection. Maximum heart rate is a hard cut off that will disregard any R waves in which the heart rate exceeds the maximum heart rate setting. Peak bias indicates whether R waves tend to deflect more positively or negatively from the baseline. A higher percent value for peak bias would instruct the software to prioritize marking the positive portion of the QRS complex as the R wave. ^*^Minimum heart rate is not a hard cut off, rather, the software will try to find a missing R wave (by lowering the QRS detection threshold) if the minimum RR is obtained and it doesn’t find an R wave.

### RR Interval Exclusion Methods

Using Data Insights software, abnormal RR intervals were marked as bad data by inserting a “bad data mark” without placing a substitute. This effectively removed the abnormal RR interval from data analysis in the Ponemah HRV analysis module. This does not affect most of the HRV parameters: SDNN, SDANN, and SDNNIDX as these measures are not affected by the order of the beats. For calculating beat-to-beat changes for rMSSD, RR intervals with a “bad data mark” in between will not be calculated. This effectively prevents including the delta change from two beats that were not normal consecutive beats.

For the manual exclusion method, as described in our previous studies (Chen et al., 2008; Pham et al., 2009), all RR intervals were visually inspected. Misplaced R wave marks were corrected by removing the misplaced mark and inserting an R wave mark at the correct location. For sections without visible R waves (masked by noise and artifacts) or where an ectopic beat occurred, a “bad data mark” is placed to mark the region as bad data. This method is most time-consuming, but it yields the most accurate data set representing normal-to-normal RR intervals.

For the % change exclusion method, two approaches were employed for this method: the “either” and the “both” approaches. The “either” approach represents the concept that the RR interval of interest is at least a defined percent change from either one of the two adjacent RR intervals. The algorithm in the Data Insights software for this exclusion approach is as follows for 20% as the cutoff criterion:

$$\begin{array}{c} \%\text{ change }({\text{RRI}}_{\text{cyc}1},{\text{ RRI}}_{\text{cyc}0})>20\\ \text{OR }\%\text{ change }({\text{RRI}}_{\text{cyc}-1},{\text{ RRI}}_{\text{cyc}0})>20 \end{array}$$

Where,

1. RRI_cyc0_ is the RR interval of interest (to be included or excluded)
2. RRI_cyc1_ is the RR interval immediately after RRI_cyc0_
3. RRI_cyc–1_ is the RR interval immediately before RRI_cyc0_

If either % change is greater than 20, RRI_cyc0_ will be marked as bad data.

The “both” approach represents the concept that the RR interval of interest is at least a defined percent change from both of the two adjacent RR intervals. The algorithm in the Data Insights software for this exclusion approach is as follows for 20% as the cutoff criterion:

$$\begin{array}{c} \%\text{ change }({\text{RRI}}_{\text{cyc}1},{\text{ RRI}}_{\text{cyc}0})>20\\ \text{AND }\%\text{ change }({\text{RRI}}_{\text{cyc}-1},{\text{ RRI}}_{\text{cyc}0})>20 \end{array}$$

Where,

1. RRI_cyc0_ is the RR interval of interest (to be included or excluded)
2. RRI_cyc1_ is the RR interval immediately after RRI_cyc0_
3. RRI_cyc–1_ is the RR interval immediately before RRI_cyc0_

Only when both of the % changes are greater than 20, will the RRI_cyc0_ be marked as bad data.

For the 2 SD exclusion method, the mean and standard deviation were calculated from the initial data set. All RR intervals outside of two standard deviations of the mean were marked as bad data and excluded from downstream HRV analysis (Thireau et al., 2008).

### Receiver Operating Characteristic (ROC)

ROC curves were used to evaluate the quality of each RR interval exclusion method for their sensitivity to correctly identify true normal-to-normal RR intervals and their specificity to correctly reject abnormal RR intervals. Four time points, each consisting of 20,000 RR intervals, from each animal were used as proof of principle for ROC analysis: Dark 1, Dark 2, Light 1, and Light 2. “Dark 1” was taken from the beginning of the dark cycle (the first 20,000 RR intervals when the light was switched off). “Dark 2” was taken from the middle of the dark cycle (20,000 RR intervals around 1:00 am during the 7pm–7am dark cycle). Likewise, “Light 1” was taken from the beginning of the light cycle (when the light was switched on) and “Light 2” was taken from the middle of the light cycle (around 1:00 pm during the 7am–7pm light cycle).

For these two approaches, the % change of the RR interval of interest from the RR interval immediately before and after it was calculated. Mathematically, the larger of these two % changes is the exclusion value for the “either” approach and the smaller of these two % changes is the exclusion value for the “both” approach.

From the initial data sets, each of the 20,000 RR intervals was visually inspected; normal RR intervals were assigned a number “1” and abnormal RR intervals were assigned a number “0.” For ROC curves based on % change, the % change of the RR interval of interest from it’s adjacent RR was calculated as follows:

$$\begin{array}{c} 100\times |{\text{RRI}}_{\text{cyc}1}-{\text{RRI}}_{\text{cyc}0}|/{\text{RRI}}_{\text{cyc}1}\\ 100\times |{\text{RRI}}_{\text{cyc}-1}-{\text{RRI}}_{\text{cyc}0}|/{\text{RRI}}_{\text{cyc}-1} \end{array}$$

For the “either” approach, the smaller of the two above % change was used for the RR interval of interest. For the “both” approach, the larger of the two above % change was used for the RR interval of interest.

For ROC curves based on SD, the following formula was used:

$$\text{F}(\text{x})=({\text{RR}}_{\text{i}}-{\text{RR}}_{\text{mean}})/\text{SD}$$

Three RR means and SDs were used: mean and SD generated from the data used to generate ROC curves (i.e., from the 20,000 RR intervals used to generate the ROC curves), from the 12 h data of the same dark-light cycle, and from the whole 24-h data set. Statistical analysis to compare ROC curves generated from all methods was performed using SigmaPlot software (Systat Software, Inc., San Jose, CA, United States). Sensitivity and specificity of different exclusion criteria were compared with a one-way repeated measure ANOVA. The Fisher LSD *post hoc* test was used for pairwise comparison when appropriate. Significance was set at *p* < 0.05.

### Generating Standard 24-h HRV Parameters

Using Data Insights software, 24-h HRV parameters were generated for the following exclusion criteria: (1) manual exclusion, (2) 15% exclusion (all RR intervals that differed by >15% from either the previous or subsequent RR intervals were excluded), (3) 20% exclusion (all RR intervals that differed by >20% from either the previous or subsequent RR intervals were excluded), (3) 1.5 SD exclusion (all RR intervals that were outside of mean ± 1.5 SD were excluded), and (4) 2 SD exclusion (all RR intervals that were outside of mean ± 2 SD were excluded). The mean and SD from the 24-h data were used for the SD exclusion method. Standard time domain HRV measures (Table 2) were generated (Malik et al., 1996).

**TABLE 2:** Time domain HRV parameters and definitions^*^.

**Parameter**	**Definition**
SDNN (ms)	Standard deviation of all RR intervals
SDANN (ms)	Standard deviation of all 2-min RR interval averages
SDNNIDX (ms)	Averages of standard deviation of all 2-min RR intervals
rMSSD (ms)	Root mean square of successive difference

^*^Normally, HRV analysis is only calculated from normal-to-normal RR intervals. For the purpose of demonstrating relative accuracy of each exclusion method, HRV parameters were generated from all data sets containing various degrees of abnormal RR intervals.

## Results

### RR Interval Composition and Distribution

**FIGURE 1:**
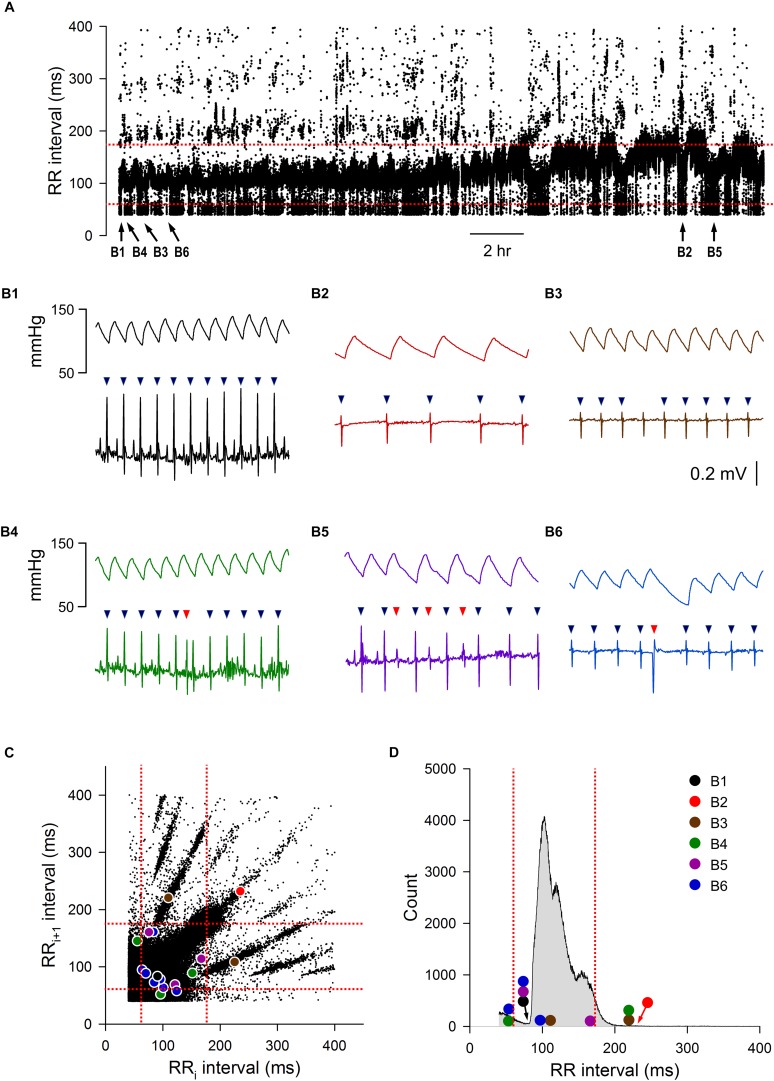
Initial data set of mouse ECG recording 1. **(A)** 24-h tachogram (RR intervals over time). The times by which the example traces in B were obtained are marked at the bottom of the tachogram. **(B)** Example traces of blood pressure and ECG recordings with the R wave marking results (triangles): normal RR interval during fast heart rate **(B1)**, normal RR interval during slow heart rate **(B2)**, an R wave that the program failed to mark, resulting in an abnormal RR interval **(B3)**, a noise that was marked as R wave (red triangle), resulting in two consecutive abnormal RR intervals **(B4)**, three extra marks (red triangles) due to noise in the recordings, resulting in six consecutive abnormal RR intervals **(B5)**, and an ectopic beat (premature ventricular contraction, red triangle) that also resulted in two consecutive abnormal RR intervals **(B6)**. **(C)** Lorenz plot of 24-h RR intervals. **(D)** Frequency histogram of the 24-h RR intervals. Color circles, RR intervals of the example traces in B. Red dotted lines, mean RR interval ± 2 SD.

An example of a 24-h tachogram obtained from a mouse is shown in Figure 1A. As expected, there were normal swings in RR intervals over the 24-h period; there were also obvious abnormal RR intervals throughout. Examples of normal and abnormal RR intervals are shown in Figure 1B. The normal-to-normal RR intervals ranged from 75 ms (Figure 1B1) to 250 ms (Figure 1B2). Abnormal RR intervals came from either failing to mark a normal R wave (Figure 1B3), errors in R wave identification (Figures 1B4,B5, red triangles), or the presence of ectopic beats/arrhythmias (Figure 1B6, red triangle). These examples were highlighted on the Lorenz plot of all 24-h of RR intervals (Figure 1C) and on the RR interval distribution histogram (Figure 1D). While some of these abnormal RR intervals were outside of the mean ± 2 SD range (defined by the red dotted lines in 1C and 1D), a significant number of abnormal RR intervals were within the 95% confidence interval. In addition, some of the longer intervals (e.g., the normal interval shown in Figure 1B2) were also outside of the 95% confidence interval and will be excluded using the 2 SD exclusion criterion.

### ROC Curve Analysis

**FIGURE 2:**
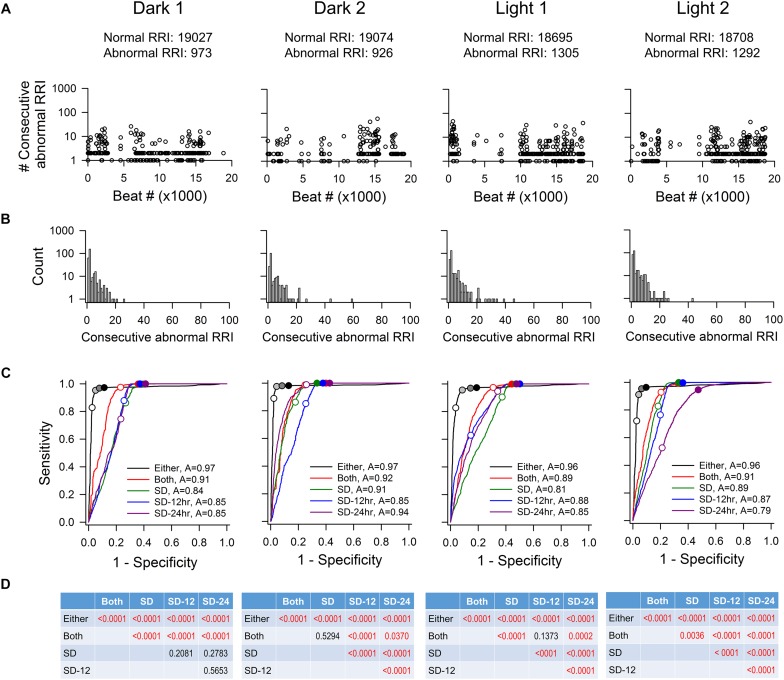
ROC analysis of four time points (20,000 RR intervals in each time point) from mouse recording 1. **(A)** Pattern of abnormal RR intervals throughout 20,000 beats. **(B)** Frequency histogram of consecutive abnormal RR intervals. Single and two consecutive abnormal RR intervals were the most frequent occurrences showing two consecutive abnormal RR intervals has the highest occurrence, following by single abnormal RR interval. **(C)** ROC curves showing that the “either” approach is significantly more accurate (highest ROC curve area) in identifying normal RR intervals with the least number of false positives. For the “either” approach, circles on the ROC curves represent 5% change (open circle), 10% change (light gray), 15% change (dark gray), and 20% change (black). For the “both” approach, open circle represents 5% change and closed circle represents 10% change. For the SD method, open circles represent 1 SDs and closed circles represent 2SDs. **(D)** *p*-values of pairwise comparison between exclusion methods. SD, SD generated from the 20,000 RR interval used for ROC analysis. SD-12 h, SD generated from the 12 h data of the same dark-light cycle. SD-24 h, SD generated from the whole 24-h data. The letter “A” in figure legend indicates overall accuracy of identifying normal and abnormal RR intervals.

To determine how accurate each method was in correctly including or eliminating RR intervals for downstream HRV analysis, four time points (Dark 1, Dark 2, Light 1, and Light 2) from each recording were taken for ROC analysis. Figure 2 shows the characteristics of abnormal RR intervals of each time point and the ROC analysis of the mouse recording in Figure 1. In this mouse, there were 926–1305 visually identified abnormal RR intervals out of the 20,000 marked RR intervals at each time point in the initial data set. The number of consecutive abnormal intervals contained was graphed to demonstrate how pervasive these intervals were throughout the each time period (Figure 2A). These abnormal RR intervals were spread throughout the recording period and appeared as a single isolated abnormal RR interval or in clusters with the number of consecutive abnormal RR intervals ranging from 2 to 47 (Figure 2A). As shown in the frequency histogram (Figure 2B), the highest occurrence of the abnormal RR interval cluster had two consecutive abnormal RR intervals, originating from either extra error marks (e.g., Figures 1B4,B5) or ectopic beats (e.g., Figure 1B6). The second highest occurrence of the abnormal RR interval cluster contained only one abnormal interval, resulting from the program failing to mark a normal R wave (e.g., Figure 1B3).

As described in the Materials and Methods, all 20,000 RR intervals were visually inspected for each time point. All normal-to-normal RR intervals were assigned to the number “1” and all abnormal RR intervals were assigned to the number “0.” Figure 2C shows the resulting ROC curves of each exclusion method. Graphically, the top-left corner of the ROC graph (where 1-specificity = 0 and Sensitivity = 1) represents a “perfect” outcome in which all normal-to-normal RR intervals are correctly identified for inclusion and all abnormal RR intervals are correctly identified for rejection. The ROC curve that is closest to this top-left corner will have the largest area under the curve and thus, the highest accuracy in identifying normal and abnormal RR intervals. The accuracy of the “either” approach of the % change method in identifying normal and abnormal RR intervals (area under the ROC curve) was between 0.96 and 0.97 (Figure 2C). For the SD exclusion method, the accuracy was between 0.79 and 0.94 (Figure 2C). There were no consistent differences among the three means ± SDs used. Results of pairwise comparison of the ROC curves are presented in Figure 2D. The “either” approach of the % change method was significantly more accurate in identifying normal and abnormal RR intervals, compared to all other ROC curves. Table 3 summarizes the range of accuracy of all methods in all animals. The “either” approach consistently had the highest accuracy. The data suggest that using % change from either adjacent RR intervals is a good method for data exclusion in mice.

**TABLE 3:** Accuracy of detecting normal and abnormal RR intervals.

	**Either**	**Both**	**SD**	**SD-12 h**	**SD-24 h**
M 1	0.96 – 0.97	0.89 – 0.92	0.81 – 0.91	0.85 – 0.88	0.79 – 0.94
M 2	0.94 – 0.98	0.85 – 0.91	0.91 – 0.98	0.88 – 0.93	0.83 – 0.93
M 3	0.92 – 0.99	0.91 – 0.97	0.88 – 0.92	0.84 – 0.93	0.81 – 0.92
R 1	0.93 – 0.98	0.93 – 0.98	0.91 – 0.97	0.91 – 0.96	0.87 – 0.93
R 2	0.93 – 0.95	0.93 – 0.95	0.90 – 0.94	0.87 – 0.93	0.83 – 0.90
R 3	0.98 – 0.99	0.93 – 0.96	0.88 – 0.92	0.83 – 0.92	0.80 – 0.88

*SD, SD generated from the 20,000 RR intervals used for ROC analysis. SD-12 h, SD generated from the 12 h data of the same dark-light cycle. SD-24 h, SD generated from the whole 24-h data. M1, mouse recording 1; M2, mouse recording 2; M3, mouse recording 3; R1, rat recording 1; R2, rat recording 2; R3, rat recording 3.*

For each ROC curve as the exclusion value increases, the sensitivity (accuracy of correctly identifying normal RR intervals) increases and the specificity (accuracy of correctly identifying abnormal RR intervals) decreases (Figure 2C). For example, an exclusion criterion of 15% change in RR interval from either adjacent RR intervals had 0.95–0.98 sensitivity with 0.86–0.94 specificity. Increasing the exclusion criterion to 20% change yielded slightly higher sensitivity (0.96–0.98) and slightly lower specificity (0.81–0.90). In contrast, increasing the exclusion value to improve sensitivity had a greater negative impact on the specificity for the SD exclusion method. Setting the exclusion level at 1 SD resulted in the sensitivity ranging from 0.47 to 0.94 with specificity ranging from 0.79 to 0.85 (Figure 2C). Increasing the exclusion value to 2 SD included nearly all normal-to-normal RR intervals (sensitivity = 0.95–1.00), but this came at the expense of a dramatic decrease in specificity (0.52–0.59).

**FIGURE 3:**
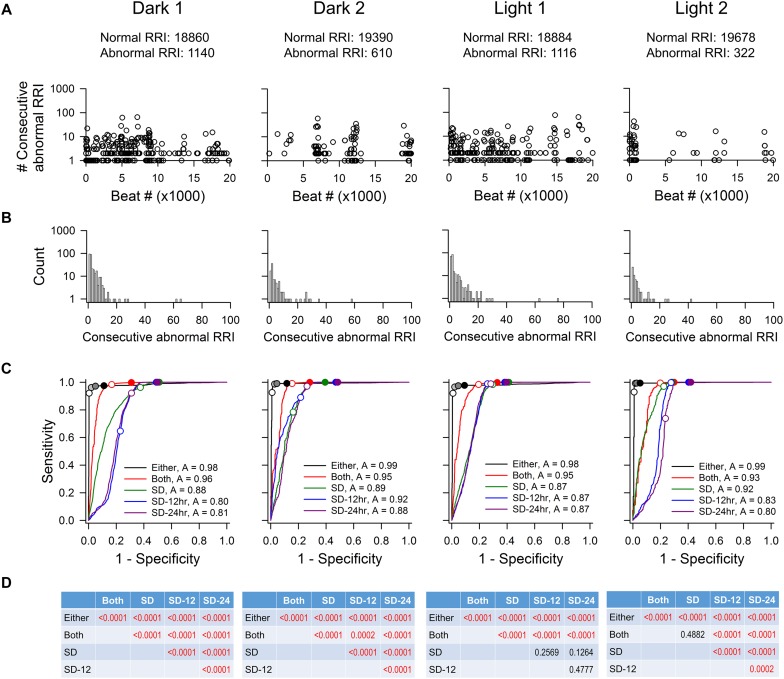
ROC analysis of four time points (20,000 RR intervals in each time point) from rat recording 3. **(A)** Pattern of abnormal RR intervals throughout 20,000 beats. **(B)** Frequency histogram of consecutive abnormal RR intervals. Single and two consecutive abnormal RR intervals were the most frequent occurrences. **(C)** ROC curves showing that the “either” approach is significantly more accurate (highest ROC curve area) in identifying normal RR intervals with the least false positives. For the “either” approach, circles on the ROC curves represent 5% change (open circle), 10% change (light gray), 15% change (dark gray), and 20% change (black). For the “both” approach, open circle represents 5% change and closed circle represents 10% change. For the SD methods, open circles represent 1 SDs and closed circles represent 2 SDs. **(D)** *p*-values of pairwise comparison between exclusion methods. SD, SD generated from the 20,000 RR interval used for ROC analysis. SD-12 h, SD generated from the 12 h data of the same dark-light cycle. SD-24 h, SD generated from the whole 24-h data. The letter “A” in figure legend indicates overall accuracy of identifying normal and abnormal RR intervals.

Figure 3 shows the characteristics of abnormal RR intervals of each time point and the ROC analysis of a rat. Out of the 20,000 marked RR intervals in each time point, the visually identified abnormal RR intervals ranged from 322 to 1140. These abnormal RR intervals appeared as a single isolated abnormal RR interval or in clusters with the number of consecutive abnormal RR intervals ranging from 2 to 76 (Figure 3A). The clusters containing one or two consecutive abnormal intervals had the highest occurrence (Figure 3B). As in mice, the ROC curve that is closest to the top-left corner is the “either” approach of the % change method (Figure 3C), indicating that the “either” approach will have the highest accuracy in identifying normal and abnormal RR intervals (overall accuracy of 0.97–0.99). This was confirmed by the pairwise comparison analysis showing a significant difference between “either” and all other methods (Figure 3D). This observation is consistent across all three rats tested (Table 3). The data suggest that the % change from either adjacent RR intervals is a good method for data exclusion in rats. Similar to the mouse data, increasing the exclusion value from 1 to 2 SD improved the sensitivity with a dramatic decrease in specificity whereas increasing the % change from 10 to 20% only had slight impact on the sensitivity and specificity.

### % Change (“Either” Approach) Versus SD

**FIGURE 4:**
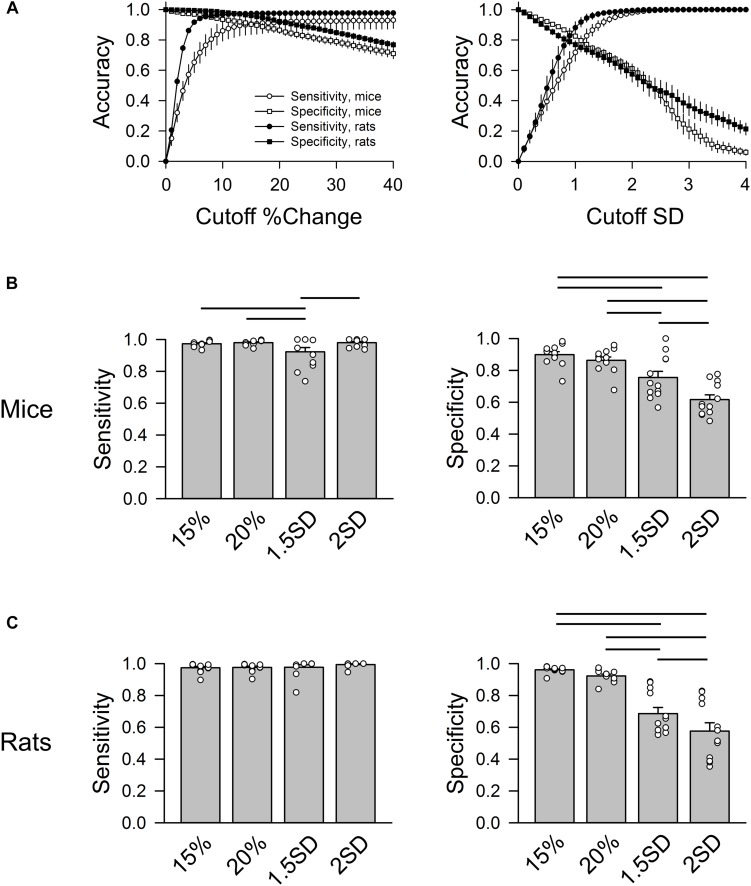
Group data of sensitivity and specificity in mice (*n* = 3) and rats (*n* = 3). **(A)** Accuracy of sensitivity (circles) and specificity (squares) at different % change cutoff values (left) and SD cutoff values (right). For % change, the sensitivity and specificity curves intersect at 10–20% change. For SD exclusion method, the sensitivity and specificity curves intersect at ∼1 SD. **(B)** Group data of four cutoff values (exclusion criteria) in mice: 15%, 20%, 1.5 SD, and 2 SD showing that 1.5 SD exclusion criterion had the lowest sensitivity. 15 and 20% had significantly higher specificity than 1.5 SD and 2 SD criteria. **(C)** Group data of four cutoff values in rats showing that 15 and 20% had significantly higher specificity than the 1.5 SD and 2 SD criteria. Horizontal bars represent significant difference (*p* < 0.05) between the two groups.

Figure 4 shows the accuracy of correctly identifying normal RR intervals (sensitivity) and abnormal RR intervals (specificity) at different % change (for the “either” approach) and SD cutoff values from all animals (Figure 4A). As the cutoff value increases, sensitivity rose exponentially while specificity decreased in a linear fashion. The intersection of the sensitivity and specificity indicates the point where the cutoff value produces the same accuracy for sensitivity and specificity. This intersection was between 10 and 20% for the “either” approach and around 1 SD for the SD-exclusion method. Setting the exclusion criterion below this point (e.g., 5% or 0.5 SD) will bias toward higher specificity at the expense of lower sensitivity. On the other hand, setting the exclusion criterion above this point (e.g., 30% or 2 SD) will bias toward higher sensitivity with lower specificity. Two % change cutoff values (15% and 20%) and two SD cutoff values (1.5 SD and 2 SD) were further compared using one-way repeated ANOVA (Figures 4B,C).

In mice (Figure 4B), 15% and 20% cutoff criteria had similar sensitivity and specificity. Both had significantly higher specificity than the 2 SD exclusion criterion. Decreasing the SD cutoff criterion from 2 to 1.5 SD significantly improved the specificity (albeit still significantly lower than the 15% and 20% cutoffs); however, this significantly reduced the sensitivity. In rats, there was no significant difference in sensitivity across the four cutoff criteria (Figure 4C). There was also no difference in specificity between 15 and 20% exclusion criteria, while 2 SD had the lowest specificity. The data suggest that 15% – 20% change from either adjacent RR intervals is a better exclusion approach than the 2 SD exclusion method.

### 24 h HRV Analysis

Time domain HRV parameters of the 24 h initial and manual exclusion data sets are presented in Table 4. The mean RR intervals were similar between the two sets of data (within 3% of each other). As expected, HRV measures generated from the initial data sets were larger than those determined after manually excluding abnormal RR intervals. Also as expected, the degree of overestimation in HRV was dependent on the degree of noise and artifact in the recordings (i.e., lower overestimates in mouse recording 3 and rat recording 1 in which the degree of noise and artifact were lower). Among the HRV measures, short-term HRV (rMSSD) was most sensitive to noise and artifacts in the recordings (e.g., 311% and 989% higher than after manual exclusion for mouse and rat, respectively), followed by SDNNIDX.

**TABLE 4:** 24-h time domain heart rate variability from initial and manual exclusion data sets.

	**RR (ms)**	**SDNN (ms)**	**SDANN (ms)**	**SDNNIDX (ms)**	**rMSSD (ms)**
M1, manual	121.5	23.33	22.65	8.10	4.28
M1, initial	118.3	27.89	23.53	15.55	17.61
	(−3%)	(20%)	(4%)	(92%)	(311%)
M2, manual	122.9	21.00	18.52	10.56	7.43
M2, initial	120.2	27.07	24.07	17.83	19.57
	(−2%)	(29%)	(30%)	(69%)	(163%)
M3, manual	111.4	19.76	17.63	9.44	5.21
M3 initial	111.0	20.32	17.70	10.40	7.15
	(−0.4%)	(3%)	(0.4%)	(10%)	(37%)
R1, manual	178.5	22.80	21.07	7.94	3.63
R1, initial	178.0	24.39	21.28	10.83	9.45
	(−0.3%)	(7%)	(1%)	(36%)	(160%)
R2, manual	185.4	22.02	21.32	8.79	5.48
R2, initial	185.1	53.74	27.77	46.26	59.65
	(−0.2%)	(144%)	(30%)	(426%)	(989%)
R3, manual	157.9	18.98	30.03	5.18	3.90
R3 initial	157.6	31.61	17.70	23.04	29.48
	(−0.2%)	(67%)	(64%)	(345%)	(656%)

*NN, mean RR interval. Number in parentheses represent % change from the manual exclusion data.*

**FIGURE 5:**
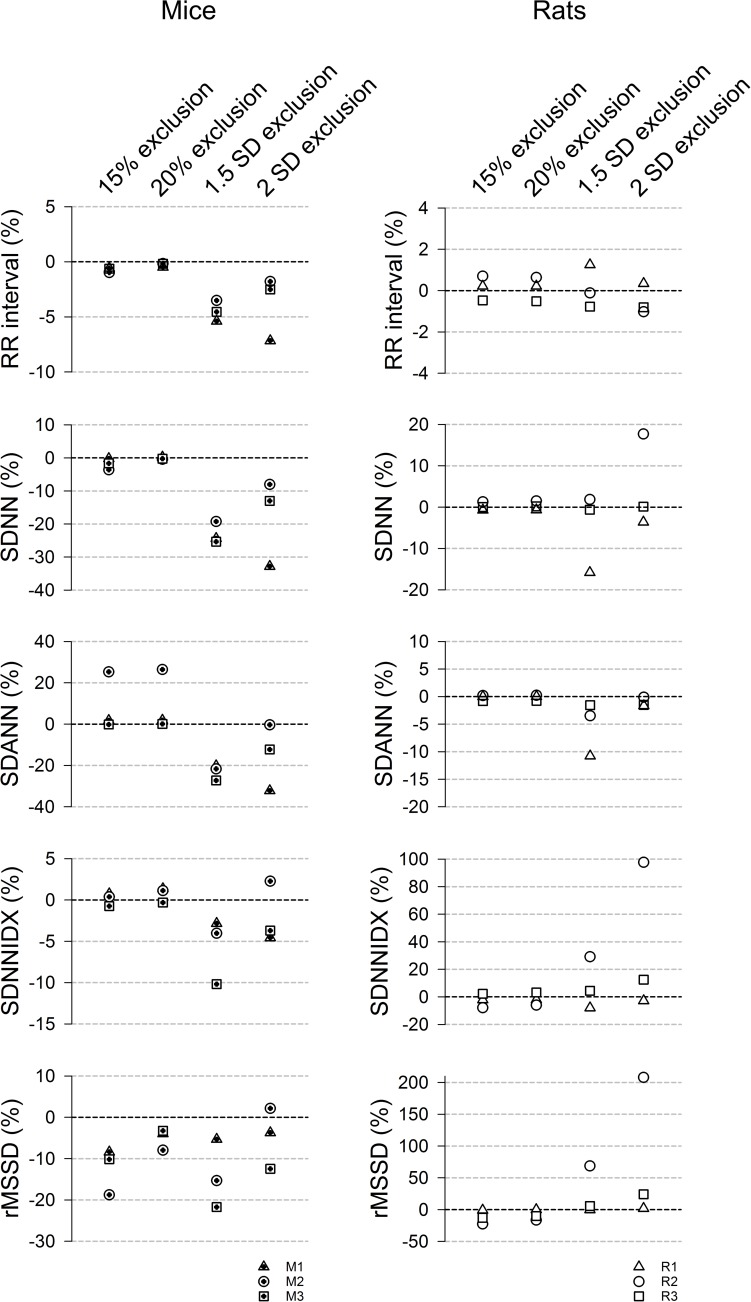
24-h time domain HRV parameters using different exclusion criteria (15%, 20%, 1.5 SD, and 2 SD) in mice (left) and rats (right). Data are expressed as % difference from manual exclusion. Overall, the HRV parameters were closer to the manual exclusion method using the % change method, compared to the SD-exclusion method. In addition, the SD methods had more variable estimates. 20% criterion had better rMSSD estimate than the 15% criterion in mice but had similar results in rats.

Standard HRV parameters were generated for two % change (15% and 20%) and two SD (1.5 and 2 SD) cutoff values and expressed as % difference of those from the manual exclusion (Figure 5). In mice (Figure 5, left panels), the “either” approach of the % change exclusion method (15% or 20%) resulted in a mean RR interval that was within 1% of the data generated using the manual exclusion method while the 2 SD exclusion consistently underestimated the mean RR interval in mice (Figure 5). For the overall HRV (SDNN), 20% exclusion yielded the closest estimate (within 0.5% of manual exclusion), while the SD exclusion method consistently underestimated the SDNN by 12% – 32%. Intriguingly, the variability in HR from 2 min cycles (SDANN) showed inconsistent results. The either approach did better than the SD method in mouse recordings 1 and 3, but worse than the SD method in mouse recording 2. This is one parameter where the pattern of noise and artifacts in the recordings made a difference in the outcome, likely due to the fact that the % change is less sensitive to long stretches of abnormal RR interval that have similar mark-to-mark intervals. However, these abnormal long intervals can be eliminated with the 2 SD exclusion method. For SDNNIDX, both 15% and 20% exclusion did slightly better than the SD exclusion. For the short term HRV measure (rMSSD), 20% exclusion consistently resulted in estimates that were within 9% of the numbers generated using manual exclusion, while 2 SD exclusion yielded inconsistent results that ranged from overestimations to underestimations by more than 12%. 15% and 1.5 SD exclusion criteria consistently underestimated the rMSSD. Together, the data suggest that using the 20% change from “either” adjacent RR intervals as an exclusion criterion is a good way for analyzing 24-h HRV in mice.

For rats (Figure 5, right panels), 15% and 20% exclusion criteria produced similar results consistently (less than 8% difference from manual exclusion for RR interval, SDNN, SDANN, and SDNNIDX). The SD exclusion criteria had inconsistent results ranging from overestimation to underestimation. For the short-term HRV (rMDSSD), the % change criteria consistently similarly underestimated the rMSSD across the three rats while the SD exclusion had variably overestimated across the three rats. Together with the ROC analysis, the data suggest that the 20% change from “either” adjacent RR intervals is a good exclusion criterion for analyzing 24-h HRV in rats.

### Visualizing Data Exclusion Methods

**FIGURE 6:**
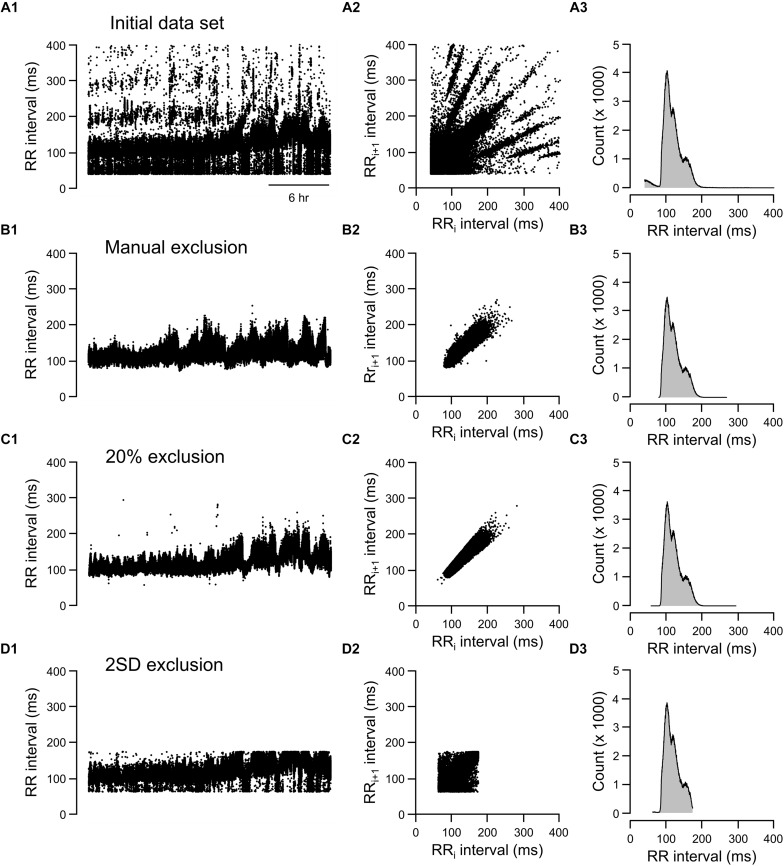
Tachograms, Lorenz plots, and frequency histograms of 24-h data from mouse recording 1 before any exclusion process (**A**, initial data set), after excluding abnormal RR intervals manually (**B**, manual exclusion), after excluding all RR intervals that were >20% different from either adjacent RR intervals (**C**, 20% exclusion), and after excluding all RR intervals that were outside of the 95% confidence intervals (**D**, 2 SD exclusion). The tachogram, Lorenz plot, and frequency histogram shown in Figure 1 are replotted here (Figure 7A) for ease of comparing the three exclusion methods (manual, 20% change, and 2 SD) to the initial data set.

**FIGURE 7:**
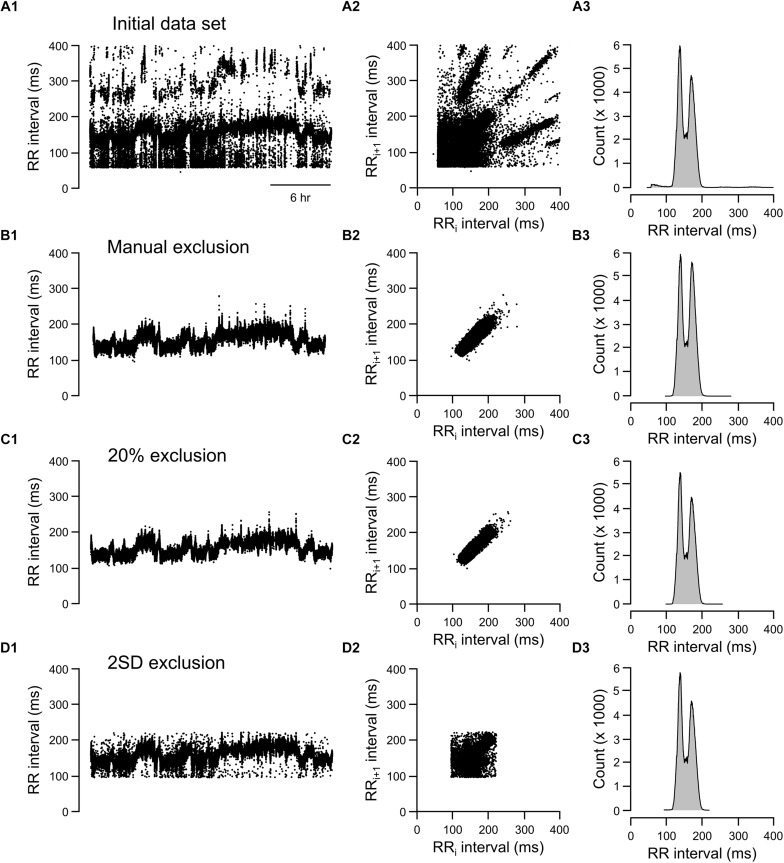
Tachograms, Lorenz plots, and frequency histograms of 24-h data from rat recording 3 before any exclusion process (**A**, initial data set), after excluding abnormal RR intervals manually (**B**, manual exclusion), after excluding all RR intervals that were >20% different from either adjacent RR intervals (**C**, 20% exclusion), and after excluding all RR intervals that were outside of the 95% confidence intervals (**D**, 2 SD exclusion).

Three graphic representations illustrating how each exclusion method affected the same 24-h data set are shown in Figures 6, 7. For mouse recording 1 (Figure 6), the 20% change from either adjacent RR intervals criterion clearly more closely resembled the manual exclusion in tachogram (Figures 6B1,C1), Lorenz plot (Figures 6B2,C2), and frequency histogram (Figures 6B3,C3) than the 2 SD exclusion method (Figures 6D1–D3). In addition, 20% change effectively excluded all examples of abnormal RR intervals shown in Figures 1B4–B6.

For the rat example (Figure 7), the 20% exclusion criterion clearly included more normal-to-normal RR intervals and excluded more abnormal RR intervals than the 2 SD method (Figures 7C,D). Overall, the graphic representations is consistent with the ROC analysis. That is, 20% change criterion had higher specificity (correctly excluded abnormal RR intervals, especially for data sets containing a higher degree of artifacts) and higher sensitivity (including normal-to-normal RR intervals, especially for data sets having relatively few artifacts).

## Discussion

The major finding of this study is that using 20% change from “either” adjacent RR intervals as the exclusion criterion can correctly identify nearly all normal-to-normal RR intervals without compromising specificity. This was possible to achieve because the majority of the incorrectly identified RR intervals and arrhythmias violated the 20% search criterion applied and few normal-to-normal RR intervals varied by more than 20% from the previous or subsequent beat. By using 20% change from “either” adjacent RR intervals as the exclusion criterion, we reliably obtained good estimates of time domain HRV measures from 24-h ECG recordings in rodents irrespective of the quality of ECG recordings. In contrast, while the 2 SD exclusion criterion had the sensitivity to include almost all normal RR intervals, it did not have the same specificity to exclude abnormal RR intervals.

An inevitable problem with long-term (24 h) recordings is that it is impossible to obtain a 24-h data set that is free of ectopic beats and artifacts. A common practice in avoiding a data set with abnormal RR intervals is to perform short-term (2–5 min) recordings for HRV analysis (Gehrmann et al., 2000; Bennett et al., 2018). While HRV measures obtained from short-term recordings provide useful clinical information (particularly regarding vagal regulation of the heart), they are not substitutes for the HRV measures obtained from complete 24-h recordings that have better correlation with clinical outcomes (Malik and Camm, 1990). For example, Malik et al. (1990) demonstrated that, in comparison to obtaining HRV from short-term recordings, assessing HRV over a 24-h period provided a better prediction of risk from arrhythmic events after an acute myocardial infarction. This may be due to the fact that long-term recordings encompass slower fluctuations in body activity/function, such as circadian rhythms, as well as physiological responses to changes in internal and external environments (e.g., reflexes, physical activity, hormones, endocrine, temperature) (Shaffer and Ginsberg, 2017).

While the clinical significance of HRV as a diagnostic comorbidity associated with numerous pathologies is well established, the mechanisms underlying the onset of attenuated HRV remain poorly understood. Rodent models of disease, especially transgenic mouse models for human diseases, can provide invaluable insights into mechanisms underlying reduced HRV and autonomic function. Presently, there is no expeditious way to accurately obtain continuous HRV from animal models, which prevents us from being able to study how subtle changes in HRV relate to disease status: As the initiating step preceding disease progression or as a symptom of worsening illness. Here, we demonstrate that the use of % change can accurately estimate HRV measures from long-term recordings in rodents. Importantly, the process significantly shortens the time required for analysis, making it feasible to process long-term recordings. By providing accurate HRV measures (reducing the required effective sample size) and significantly shortening the processing time, this method can increase the throughput of data analysis by eliminating the bottleneck associated with manual identification and exclusion. Consequently, this methodology could facilitate the use of rodent models in our understanding of mechanisms underlying reduced HRV.

Key to using animal models for studying underlying mechanisms for human disease is that the mechanism of neural regulation of HRV is similar between humans and chosen animal models. We and others have shown that, even though resting HR in mice is chiefly under cardiac sympathetic influence (unlike in humans in which resting HR is chiefly under cardiac vagal influence), time domain short-term HRV (rMSSD) is mostly under cardiac vagal regulation just as it is in humans (Gehrmann et al., 2000; Pham et al., 2009). Similarly, the corresponding HRV measures in frequency domain (high frequency power) has been shown to be under cardiac vagal regulation, just as in humans (Tsai et al., 2012).

While it is well accepted that HRV is a strong, independent risk factor for several cardiovascular maladies, including arrhythmias and sudden cardiac death (Kleiger et al., 2005; Billman, 2011), experts in the field have yet to identify a numerical value or threshold beneath which HRV values are deemed “pathological” (Buccelletti et al., 2009). Unlike BP and HR where the normal range is well defined and consistent across subjects, HRV values can vary from subject to subject (Sato et al., 1995; Gregoire et al., 1996; Melanson, 2000). In addition, the observed changes in HRV under different conditions are often less than 20% (Buccelletti et al., 2009; Pieters et al., 2012). Given the overlap in HRV values between normal and altered physiological states (Rajendra Acharya et al., 2006; Benichou et al., 2018; Poliwczak et al., 2018), our proposed data exclusion method is likely to be applicable to most physiological/pathological conditions. Furthermore, our results showed that % change exclusion criterion is a feasible way for analyzing long-term recordings, providing a basis for future studies looking at pathological conditions. It is possible that the absolute % change may differ from study to study. As proof of principle, we showed that the use of ROC analysis could provide a useful tool for evaluating the appropriate cutoff value for % change and the accuracy of the exclusion method. This analysis could be easily applied to future studies for better estimate of HRV measures.

Here, we verified that a 20% change from “either” adjacent RR intervals as an exclusion criterion is a good approach for analyzing HRV in rodents. This approach significantly reduced the time required to exclude abnormal RR intervals for HRV analysis from 1 week (manual exclusion) to less than 1 h for a 24-h data set, thereby making higher throughput analysis of 24-h HRV from rodents feasible. Similar approaches have been used in prior studies in humans, albeit without justification and verification. For example, Kleiger et al. (1987) used the criterion that the RR intervals were within 20% of the preceding RR interval as the first screening method in identifying normal RR intervals, followed by visual inspection. However, the majority of human studies with 24-h Holter recordings rely solely on visual inspection and human editing prior to HRV analysis (Gac and Sobieszczanska, 2014; Bobkowski et al., 2017). The 24-h Holter recordings are likely to have higher quality of ECG signals than those in rodent recordings. In addition, the number of heart beats in 24 h is 10-fold lower in humans than in mice. Although these factors makes it more feasible to manually exclude abnormal RR intervals for HRV analysis in humans, our results may also be useful for decreasing the processing time for 24-h Holter recordings. Kleiger et al. (1987) applied the 20% exclusion only to the RR intervals that were >20% different from the preceding RR interval as the first step and the data still went through visual inspection after the first cut. It is conceivable that excluding the RR intervals that are >20% change from either adjacent could provide enough sensitivity and specificity and eliminate the need for visual inspection. In addition, the optimal % change for exclusion could be tested with ROC analysis.

In addition to the significant advantage of being able to identify and exclude abnormal RR intervals residing within the normal RR interval range, the % change exclusion method also provides an advantage over any numerical cut-off because it is not influenced by changes in the duration of recording, the range of HR, or absolute RR interval duration. Many factors can change the range of RR intervals in the initial data set, including circadian rhythms (longer RR intervals during light cycle and shorter RR intervals during dark cycle), the animal’s activity levels, and errors in marking R waves. A larger RR interval range will result in a larger 95% confidence interval, and hence, an increased likelihood of including more abnormal RR intervals resulting in overestimation of HRV measures. On the other hand, with a relatively artifact-free initial data set, up to 5% of the normal RR intervals may be excluded with the numerical cut off criterion resulting in underestimation of HRV measures. In this regard, while HRV is an important clinical index of cardiac autonomic health, the effect size of changes in HRV may be small. For example, exposure to air pollution has been shown to reduce short-term HRV by 10–15% in humans and mice (Pope et al., 2001; Chen et al., 2008; Pham et al., 2009; Garza et al., 2016). Therefore, overestimation of HRV in some datasets and underestimation in others could mask subtle, albeit meaningful, physiological responses to exposures or under different pathology conditions.

There is, however, a potential limitation in the % change method in that it is not as sensitive to long stretches of abnormal RR intervals with similar inter-beat intervals. This limitation could explain the overestimation of SDANN for mouse recording 2. Despite this potential limitation on one HRV measure, the 20% exclusion criterion was still significantly more accurate than then that SD method in identifying normal and abnormal RR intervals in the ROC analysis. Thus, it is a good way to effectively and accurately obtain HRV measures from 24-h recordings in mice, especially for the more commonly used clinical measures (SDNN and rMSSD). Although beyond the scope of this study, this exclusion strategy may need to be modified for applying to pathological conditions. As more data are obtained using animal models of disease, we will better be able to refine how we process large quantities of data by identifying scenarios in which these methods may need modification in order to capture physiologically relevant data.

Using percent change to exclude ectopic beats, noise and artifacts from continuous ECG waveforms has proven to be a reliable and efficient way to generate accurate measures of HRV. However, this method cannot work if the R-waves in the ECG file are not sufficiently marked. Therefore, the ability to use this method is limited by how thoroughly R-waves are identified; if QRS complexes are not captured, the software cannot accurately determine the duration by which adjacent beats differ from each other. We found the Minimum R Deflection attribute to be the most important for catching waveforms of varying amplitude and shape. To improve our ability to catch all R-waves, despite variations in amplitude, shape and directionality, we conducted R wave marking over smaller regions of recording, adjusting the value for R Deflection until most R-waves were identified. Additionally, even though we recorded BP in combination with ECG as an independent measure of normal cardiac contractions for this study, BP recordings are not necessary for processing HRV with ECG signals. Considering the cost of these transmitter, ECG only transmitters is a more cost effective option for measuring HRV.

## Conclusion

While the clinical significance of HRV as a diagnostic comorbidity associated with numerous pathologies is well established, the mechanisms underlying the onset of attenuated HRV remain poorly understood. We validated the use of a % change method for excluding abnormal RR intervals as a reliable method for generating accurate HRV parameters, even when the original ECG file is rife with RR intervals that violate the normal-to-normal criterion required for HRV analysis. This method dramatically improves the efficiency of data processing of ECG-derived HRV parameters. The promising nature of these findings is exciting, and will undoubtedly open doors for research enabling us to probe how environmental, pathological, and pharmacological changes influence HRV. Applying a 20% change from either adjacent RR intervals exclusion method will enable researchers to take advantage of available rodent models to help facilitate research on the underlying mechanisms mediating reduced HRV in pathological conditions as well as allow us to better understand how changes in HRV influence physiology.

## Ethics Statement

All protocols were approved by the University of California, Davis Institutional Animal Care and Use Committee in compliance with the Animal Welfare Act and Public Health Service Policy on Humane Care and Use of Laboratory Animals.

## Author Contributions

EK and C-YC contributed to the conception and design of the study. EK, SP, AM, and C-YC organized the data and performed the statistical analysis. DB, AM, and PL contributed to the experiments involving the rats. EK wrote the first draft of the manuscript. SP, DB, PL, and C-YC wrote the sections of the manuscript. All authors contributed to manuscript revision, read and approved the submitted version.

## Conflict of Interest Statement

The authors declare that the research was conducted in the absence of any commercial or financial relationships that could be construed as a potential conflict of interest.

## References

[B1] Benichou T. Pereira B. Mermillod M. Tauveron I. Pfabigan D. Maqdasy S. (2018). Heart rate variability in type 2 diabetes mellitus: a systematic review and meta-analysis. *PLoS One* 13:e0195166. 10.1371/journal.pone.0195166 29608603 PMC5880391

[B2] Bennett B. A. Spannhake E. W. Rule A. M. Breysse P. N. Tankersley C. G. (2018). The acute effects of age and particulate matter exposure on heart rate and heart rate variability in mice. *Cardiovasc. Toxicol.* 18 507–519. 10.1007/s12012-018-9461-3 29774517 PMC13069924

[B3] Billman G. E. (2011). Heart rate variability - a historical perspective. *Front. Physiol.* 2:86. 10.3389/fphys.2011.00086 22144961 PMC3225923

[B4] Bobkowski W. Stefaniak M. E. Krauze T. Gendera K. Wykretowicz A. Piskorski J. (2017). Measures of heart rate variability in 24-h ECGs depend on age but not gender of healthy children. *Front. Physiol.* 8:311. 10.3389/fphys.2017.00311 28572771 PMC5435822

[B5] Buccelletti E. Gilardi E. Scaini E. Galiuto L. Persiani R. Biondi A. (2009). Heart rate variability and myocardial infarction: systematic literature review and metanalysis. *Eur. Rev. Med. Pharmacol. Sci.* 13 299–307. 19694345

[B6] Chen C. Y. Chow D. Chiamvimonvat N. Glatter K. A. Li N. He Y. (2008). Short-term secondhand smoke exposure decreases heart rate variability and increases arrhythmia susceptibility in mice. *Am. J. Physiol. Heart Circ. Physiol.* 295 H632–H639. 10.1152/ajpheart.91535.2007 18552155 PMC2519230

[B7] Gac P. Sobieszczanska M. (2014). Effects of cigarette smoke on Holter ECG recordings in patients with arterial hypertension. part 1: time domain parameters of heart rate variability. *Environ. Toxicol. Pharmacol.* 37 404–413. 10.1016/j.etap.2013.12.014 24444697

[B8] Garza J. L. Mittleman M. A. Zhang J. Christiani D. C. Cavallari J. M. (2016). Time course of heart rate variability response to PM2.5 exposure from secondhand smoke. *PLoS One* 11:e0154783. 10.1371/journal.pone.0154783 27223894 PMC4880193

[B9] Gehrmann J. Hammer P. E. Maguire C. T. Wakimoto H. Triedman J. K. Berul C. I. (2000). Phenotypic screening for heart rate variability in the mouse. *Am. J. Physiol. Heart Circ. Physiol.* 279 H733–H740. 10.1152/ajpheart.2000.279.2.H733 10924073

[B10] Gregoire J. Tuck S. Yamamoto Y. Hughson R. L. (1996). Heart rate variability at rest and exercise: influence of age, gender, and physical training. *Can. J. Appl. Physiol.* 21 455–470. 8959312 10.1139/h96-040

[B11] Hedman A. E. Hartikainen J. E. Tahvanainen K. U. Hakumaki M. O. (1995). The high frequency component of heart rate variability reflects cardiac parasympathetic modulation rather than parasympathetic ‘tone’. *Acta Physiol. Scand.* 155 267–273. 10.1111/j.1748-1716.1995.tb09973.x 8619324

[B12] Katona P. G. Jih F. (1975). Respiratory sinus arrhythmia: noninvasive measure of parasympathetic cardiac control. *J. Appl. Physiol.* 39 801–805. 10.1152/jappl.1975.39.5.801 1184518

[B13] Kleiger R. E. Miller J. P. Bigger J. T. Jr. Moss A. J. (1987). Decreased heart rate variability and its association with increased mortality after acute myocardial infarction. *Am. J. Cardiol.* 59 256–262. 3812275 10.1016/0002-9149(87)90795-8

[B14] Kleiger R. E. Stein P. K. Bigger J. T. Jr. (2005). Heart rate variability: measurement and clinical utility. *Ann. Noninvas. Electrocardiol.* 10 88–101. 10.1111/j.1542-474X.2005.10101.x 15649244 PMC6932537

[B15] Lippman N. Stein K. M. Lerman B. B. (1994). Comparison of methods for removal of ectopy in measurement of heart rate variability. *Am. J. Physiol.* 267(1 Pt 2), H411–H418. 10.1152/ajpheart.1994.267.1.H411 7519408

[B16] Malik M. Bigger J. T. Camm A. J. Kleiger R. E. Malliani A. Moss A. J. (1996). Heart rate variability: standards of measurement, physiological interpretation and clinical use. Task Force of the European Society of Cardiology and the North American Society of Pacing and Electrophysiology. *Circulation* 93 1043–1065. 8598068

[B17] Malik M. Camm A. J. (1990). Significance of long term components of heart rate variability for the further prognosis after acute myocardial infarction. *Cardiovasc. Res.* 24 793–803. 2085834 10.1093/cvr/24.10.793

[B18] Malik M. Farrell T. Camm A. J. (1990). Circadian rhythm of heart rate variability after acute myocardial infarction and its influence on the prognostic value of heart rate variability. *Am. J. Cardiol.* 66 1049–1054. 2220630 10.1016/0002-9149(90)90503-s

[B19] Melanson E. L. (2000). Resting heart rate variability in men varying in habitual physical activity. *Med. Sci. Sports Exerc.* 32 1894–1901. 11079519 10.1097/00005768-200011000-00012

[B20] Peltola M. A. (2012). Role of editing of R-R intervals in the analysis of heart rate variability. *Front. Physiol.* 3:148. 10.3389/fphys.2012.00148 22654764 PMC3358711

[B21] Pham H. Bonham A. C. Pinkerton K. E. Chen C. Y. (2009). Central neuroplasticity and decreased heart rate variability after particulate matter exposure in mice. *Environ. Health Perspect.* 117 1448–1453. 10.1289/ehp.0900674 19750112 PMC2737024

[B22] Pieters N. Plusquin M. Cox B. Kicinski M. Vangronsveld J. Nawrot T. S. (2012). An epidemiological appraisal of the association between heart rate variability and particulate air pollution: a meta-analysis. *Heart* 98 1127–1135. 10.1136/heartjnl-2011-301505 22628541 PMC3392690

[B23] Poliwczak A. R. Waszczykowska E. Dziankowska-Bartkowiak B. Kozirog M. Dworniak K. (2018). The use of heart rate turbulence and heart rate variability in the assessment of autonomic regulation and circadian rhythm in patients with systemic lupus erythematosus without apparent heart disease. *Lupus* 27 436–444. 10.1177/0961203317725590 28795655

[B24] Pope C. A. III Eatough D. J. Gold D. R. Pang Y. Nielsen K. R. Nath P. (2001). Acute exposure to environmental tobacco smoke and heart rate variability. *Environ. Health Perspect.* 109 711–716. 10.1289/ehp.01109711 11485870 PMC1240375

[B25] Rajendra Acharya U. Paul Joseph K. Kannathal N. Lim C. M. Suri J. S. (2006). Heart rate variability: a review. *Med. Biol. Eng. Comput.* 44 1031–1051. 10.1007/s11517-006-0119-0 17111118

[B26] Sato N. Miyake S. Akatsu J. Kumashiro M. (1995). Power spectral analysis of heart rate variability in healthy young women during the normal menstrual cycle. *Psychosom. Med.* 57 331–335. 7480562 10.1097/00006842-199507000-00004

[B27] Shaffer F. Ginsberg J. P. (2017). An overview of heart rate variability metrics and norms. *Front. Public Health* 5:258. 10.3389/fpubh.2017.00258 29034226 PMC5624990

[B28] Thireau J. Zhang B. L. Poisson D. Babuty D. (2008). Heart rate variability in mice: a theoretical and practical guide. *Exp. Physiol.* 93 83–94. 10.1113/expphysiol.2007.040733 17911354

[B29] Tsai M. L. Chen C. C. Yeh C. J. Chou L. M. Cheng C. H. (2012). Frequency ranges of heart rate variability related to autonomic nerve activity in the mouse. *Clin. Exp. Hypertens* 34 182–190. 10.3109/10641963.2011.577492 21967028

[B30] Villareal R. P. Liu B. C. Massumi A. (2002). Heart rate variability and cardiovascular mortality. *Curr. Atheroscler. Rep.* 4 120–127. 11822975 10.1007/s11883-002-0035-1

